# Red blood transfusion is a predictor of poor outcome in pediatric cardiac surgery

**DOI:** 10.1186/cc11050

**Published:** 2012-03-20

**Authors:** C Colognesi, R Maia, L Hajjar, F Galas

**Affiliations:** 1 Heart Institute, São Paulo, Brazil

## Introduction

Red blood cell transfusion is associated with morbidity and mortality among adults undergoing cardiac surgery. We aimed to evaluate the association of transfusion with morbidity in pediatric cardiac surgical patients. The purpose of this study was to assess whether red blood cell transfusions result in worse outcomes after cardiac surgery in pediatric patients.

## Methods

We studied an observational and prospective cohort of 200 patients undergoing cardiac surgery for congenital heart disease. We recorded baseline characteristics, RACHS-1 score, intraoperative data, cardiopulmonary bypass length, type of surgery, transfusion requirement and postoperative complications as need for reoperation, time of mechanical ventilation, cardiovascular complications, acute renal failure, infection, readmission at ICU and death during 28 days.

## Results

One hundred and twenty-four patients were exposed to blood components. Seventy-seven percent of patients presented at least one major complication. There was no difference between transfused and nontransfused patients regarding baseline or intraoperative characteristics. Transfused patients presented a higher incidence of major complications than nontransfused patients (93.5% vs. 54.5%, *P *= 0.002). In a multivariate analysis, red blood cell transfusion was an independent risk factor for clinical complications including death in 28 days (OR = 2.2 (95% CI 1.4 to 23.4)).

## Conclusion

Blood transfusion after pediatric cardiac surgery is a risk factor for worse outcome. Avoiding blood transfusion may reduce mortality in this population.

